# The Overlapping Community Structure of Structural Brain Network in Young Healthy Individuals

**DOI:** 10.1371/journal.pone.0019608

**Published:** 2011-05-06

**Authors:** Kai Wu, Yasuyuki Taki, Kazunori Sato, Yuko Sassa, Kentaro Inoue, Ryoi Goto, Ken Okada, Ryuta Kawashima, Yong He, Alan C. Evans, Hiroshi Fukuda

**Affiliations:** 1 Department of Nuclear Medicine and Radiology, Institute of Development, Aging and Cancer, Tohoku University, Sendai, Japan; 2 Division of Developmental Cognitive Neuroscience, Institute of Development, Aging and Cancer, Tohoku University, Sendai, Japan; 3 Department of Functional Brain Imaging, Institute of Development, Aging and Cancer, Tohoku University, Sendai, Japan; 4 State Key Laboratory of Cognitive Neuroscience and Learning, Beijing Normal University, Beijing, China; 5 McConnell Brain Imaging Centre, Montreal Neurological Institute, McGill University, Montreal, Quebec, Canada; Indiana University, United States of America

## Abstract

Community structure is a universal and significant feature of many complex networks in biology, society, and economics. Community structure has also been revealed in human brain structural and functional networks in previous studies. However, communities overlap and share many edges and nodes. Uncovering the overlapping community structure of complex networks remains largely unknown in human brain networks. Here, using regional gray matter volume, we investigated the structural brain network among 90 brain regions (according to a predefined anatomical atlas) in 462 young, healthy individuals. Overlapped nodes between communities were defined by assuming that nodes (brain regions) can belong to more than one community. We demonstrated that 90 brain regions were organized into 5 overlapping communities associated with several well-known brain systems, such as the auditory/language, visuospatial, emotion, decision-making, social, control of action, memory/learning, and visual systems. The overlapped nodes were mostly involved in an inferior-posterior pattern and were primarily related to auditory and visual perception. The overlapped nodes were mainly attributed to brain regions with higher node degrees and nodal efficiency and played a pivotal role in the flow of informa- tion through the structural brain network. Our results revealed fuzzy boundaries between communities by identifying overlapped nodes and provided new insights into the understanding of the relationship between the structure and function of the human brain. This study provides the first report of the overlapping community structure of the structural network of the human brain.

## Introduction

**Figure 1:**
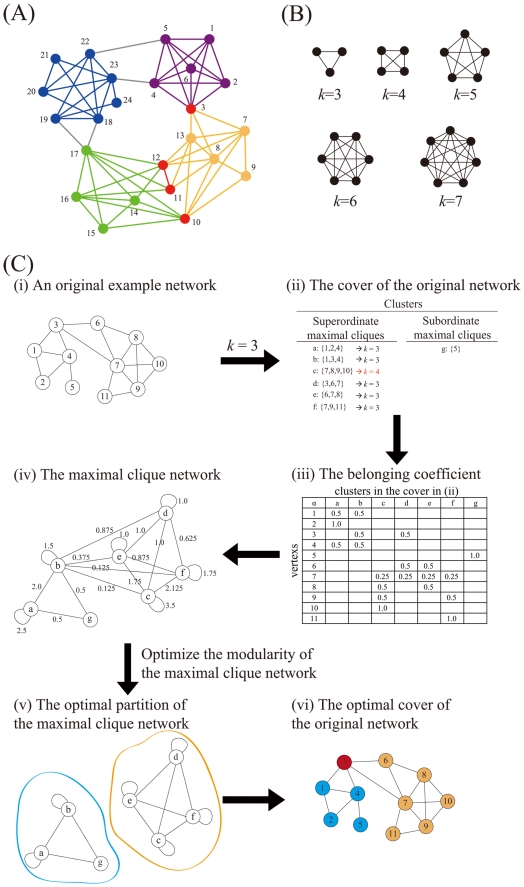
The explanation for an overlapping community structure. (A) A schematic network with overlapping communities. Communities are represented by different colors. Overlapped nodes shared by more than one community are emphasized in red. Connections between communities are shown by gray lines. (B) The definition of a clique. A clique (*k*-clique) is a complete subgraph of size *k*. (C) The flowchart for the process of determining an overlapping community structure. In the cover (ii) of the original example network (i), each superordinate maximal clique (*k* = 3, 4) is a cluster, and each subordinate maximal clique (*k*<3) forms a cluster consisting of only one vertex (here, *k* is set to 3 in the calculation). The maximal clique with *k* = 4 (red arrow) is not a subset of any other clique. The maximal clique network (iv) is obtained according to the belonging coefficient α (iii) of each vertex (1–11) to its corresponding clusters (a–g). The optimal partition of the maximal clique network (v) is computed by an efficient modularity optimization algorithm, and it can be mapped to the optimal cover of the original network (vi). In the cover (vi), it holds the information about the overlapping community structure of the original network; overlapping communities are represented by different colors, and an overlapped node is emphasized in red. Some parts of this figure are reproduced, with permission, from Shen et al., 2009b ©2009, IOP Publishing and SISSA.

Community structure is thought to be one of the main organizing principles in most complex networks, including biological, social, and economic systems [1], [2], [3], [4]. Communities or modules are groups of nodes forming tightly connected units that are only weakly linked to each other; they reflect topological relationships between elements of the underlying system and represent functional entities [5], [6]. The community structure is interpreted in terms of separated communities, whereas most real networks are also characterized by well-defined statistics of overlapping communities [6], [7], [8], [9]. A schematic network with overlapping communities is shown in Figure 1A. Overlapping community structure means that a node can belong to more than one community, which results in overlapping communities [10]. For instance, as humans, we each belong to numerous communities related to our social activities or personal lives (school, profession, friends, family, and hobbies). An extremely complicated web of our communities develops because members of our communities also belong to other communities. Overlapping community structure has been widely studied in many real-world networks, such as Zachary's karate club network, the word association network, the scientific collaboration network, Lusseau's dolphins' social network, and the molecular biology network of protein-protein interactions [5], [6], [9], [10], [11]. However, characterization of an overlapping community structure in the human brain network has not been investigated.

The features of functional and structural networks in the human brain have been well-defined; these features include small-world topology, highly connected hubs, and modularity [12], [13], [14]. The large-scale data of human brain connectivity offers the opportunity to understand the links between brain structure and function at the regional level [15]. For example, the characterization of community structure in the human brain network contributes to the identification of the anatomical and functional structures of brain regions associated with specific biological functions [16], [17], [18], [19], [20]. Some primary brain functions (e.g., motor, auditory, and visual systems) have been regularly detected in these previous studies, and significant differences in the modular organization of brain networks have also been observed. The main reasons for these differences between studies might be the different neuroimaging modalities (e.g., functional, structural, and diffusion MRI) and the characteristics of the sample of research subjects. In addition to these studies, current theories on brain organization suggest that cognitive functions, such as attention, language, and memory, are organized into widespread, segregated, and overlapping networks [21]. Moreover, some cortical areas are heteromodal; they are not restricted to any single motor or sensory function, but receive convergent information from multiple sensory and motor areas of the brain. For example, although 50% of over 200 cells in the superior temporal sulcus (STS) of anesthetized monkeys are unimodal (meaning that they respond to only one of the three sensory modalities: visual, auditory, and somatosensory), over 20% of them are bimodal or trimodal and respond to two or three modalities, respectively [22]. A previous study has identified the cortical areas that are responsive to transitions within a single sensory modality and a cortical network that is responsive to transitions in multiple sensory modalities; this study has revealed a distributed, multimodal network for involuntary attention to events in the sensory environment [23]. An attempt has been made to reveal overlapping communities in the network of the macaque monkey's visuotactile coretex; the authors have found that several areas (e.g., 46, VIP, LIP, 7a, and V4) are bridge nodes (overlaps between the visual and the somatosensory cortex), which play higher-level roles and integrate cognitive functions (e.g., attention and working memory) [24]. One may speculate that a brain region could be involved in several brain systems, and therefore, we hypothesized that such a region can be defined as an overlapped node shared by different communities in the human brain network. Thus, this study represents an interesting and challenging approach to clarify the overlapping community structure in the human brain network and would improve our understanding of how functional brain states are associated with their structure.

The main objective of this study was to reveal an overlapping community structure of the structural brain network in young, healthy individuals using regional gray matter volume (RGMV). Study participants were selected from a large-scale brain MRI database of normal Japanese people (462 subjects, ages 21 to 39 years) [25]. A structural brain network can be abstracted from human MRI data by compiling a matrix of correlations from morphological measurements (cortical thickness, RGMV, and surface area) between all pairs of regions in some parcellation scheme and then applying a threshold to create a graph representing strong (suprathreshold) correlations to connect regions [26], [27], [28]. In this study, the structural connectivity of the human brain consisting of 90 regions was constructed by computing the correlation matrix of RGMV across the population, as described in our previous study [29]. A binarized and undirected network in the human brain was then obtained by thresholding the correlation matrix with a cost threshold strategy. We identified 5 overlapping communities in the structural brain network and discovered brain functions that were involved in overlapping communities and were related to overlapped nodes. Finally, we analyzed regional nodal properties and the importance of overlapped nodes in terms of node degree, nodal efficiency, node betweenness, and the participation coefficient.

## Methods

### Ethics Statement

In accordance with the Declaration of Helsinki (1991), written informed consent was obtained from every subject and his/her parent after a full explanation of the purposes and procedures of the study was provided. Approval for these experiments was obtained from the institutional review board of Tohoku University.

### Participants

In this study, we collected brain images of 462 young, healthy subjects from a database of normal Japanese individuals [25]. The female to male ratio was 218∶244, the mean age ± S.D was 28.45±6.04 years, and the age range was 20 to 39 years. The MR images were inspected by 2 to 3 well-trained radiologists, and images with the following findings were excluded from this study: head injuries, brain tumors, hemorrhages, major and lacunar infarctions, and moderate to severe white matter hyperintensities. We did not exclude images with mild, spotty white matter hyperintensities.

### MRI acquisition

Brain images were obtained using two 0.5 T MR scanners (Sigma contour, GE-Yokogawa Medical Systems, Tokyo) with two different pulse sequences: (1) 124 contiguous, 1.5 mm thick axial planes of three-dimensional T1-weighted images (spoiled gradient recalled acquisition in steady state: repetition time (TR), 40 ms; echo time (TE), 7 ms; flip angle (FA), 30°; voxel size, 1.02 mm×1.02 mm×1.5 mm); and (2) 63 contiguous, 3 mm thick axial planes of gapless (using interleaving) proton density-weighted/T2-weighted images (dual echo fast spin echo: TR, 2860 ms; TE, 15/120 ms; voxel size, 1.02 mm×1.02 mm×3 mm). T1 images were used for the present analysis, and all three images were used to exclude MRIs with abnormalities, as described above.

### Measurements of regional gray matter volume

Following image acquisition, the RGMV for each subject was measured using statistical parametric mapping 2 (SPM2) (Wellcome Department of Cognitive Neurology, London, UK) [30] in Matlab (Math Works, Natick, MA). First, T1-weighted MR images were transformed to the same stereotactic space by registering each of the images to the ICBM 152 template (Montreal Neurological Institute, Montreal, Canada), which approximates the Talairach space [31]. Then tissue segmentation from the raw images to the gray matter, white matter, cerebrospinal fluid space, and non-brain tissue was performed using the SPM2 default segmentation procedure. WFU PickAtlas software was employed to label the regions in the gray matter images, which provided a method for generating ROI masks based on the Talairach Daemon database [32], [33], [34]. To calculate the RGMV for each subject, we divided the entire gray matter into 45 separate regions for each hemisphere (90 regions in total, see Supplementary, Table S1), as defined by the Automated Anatomical Labeling (AAL) atlas [35].

### Construction of the structural brain network

It has been well documented that there are correlated changes in gray matter morphology (e.g., cortical thickness and volume) between various anatomically or functionally linked areas. The concept of morphological correlations has been widely used to study correlated evolution in mammalian brain structures [36]. A large-scale anatomical network of the human cerebral cortex [28] was first investigated using cortical thickness measurements, which are known to be strongly correlated between regions that are axonally connected [37]. This approach has also been used to study structural brain networks in health and disease [16], [26], [27], [29], [38], [39], [40]. In this study, we used this methodology to construct a structural brain network using the RGMV measurements. First, we performed a linear regression on the RGMV of 90 regions, removing the effects of age, gender, age-gender interaction, and total gray matter volume. The residuals of this regression were substituted for the raw RGMV and were denoted as corrected RGMV (cRGMV). Secondly, we computed the Pearson correlation coefficient between the cRGMV across the 462 subjects to construct the interregional correlation matrix (*N×N*, where *N* is the number of gray matter regions; here *N* = 90). Thirdly, the correlation matrix can be converted to a binarized and undirected network *G* using a cost threshold, which is equivalent to the ratio between the number of edges and all possible edges [41].

### Detecting overlapping community structure

A clique (*k*-clique) (Figure 1B) is a complete subgraph of size *k* in which every vertex is adjacent to every other vertex [6]. A maximal clique is a clique that is not a subset of any other clique in a graph [11]. By assuming that a maximal clique only belongs to one community because of its high connectivity, overlaps between communities are allowed. The flowchart for the process of determining overlapping community structure in an example network is shown in Figure 1C. Firstly, the cover was defined as a set of clusters in an original example network. Each vertex in the original network was assigned to at least one cluster. Among the clusters in the cover, maximal cliques with a size greater than or equal to *k* were defined as superordinate maximal cliques, and those with a size smaller than *k* were defined as subordinate maximal cliques. Secondly, each cluster becomes a vertex in the resulting maximal clique network, which was defined as a weighted network by introducing the concept of the belonging coefficient of each vertex [8]. Thirdly, a partition of the maximal clique network can be mapped into a cover of the original network, which may hold the information about the overlapping community structure of the original network. We obtained the optimal cover of the original network by optimizing the quality of a cover (*Q_c_*) formalized as: [11]

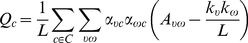
(1)


In equation (1), *A* is the adjacency matrix of the network *G*, 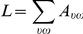 is the total weight of all edges, and 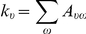 is the degree of the vertex 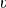. Moreover, 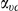 is a belonging coefficient defined in equation (2), which reflects how much the vertex 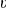 belongs to the community *c* [8].
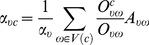
(2)


In equation (2), 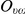 denotes the number of maximal cliques in the whole network containing the edge 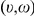, 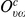 denotes the number of maximal cliques containing the edge 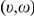 in the community *c*, and 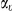 is a normalization term denoted in equation (3).
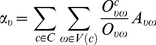
(3)


It has been demonstrated that the optimization of *Q_c_* in the original network is equivalent to the optimization of the Newman's modularity in the maximal clique network [11]. Thus, the optimal cover with overlapping communities of the original network can be identified through partitioning the maximal clique network using a fast unfolding algorithm on the modularity optimization [42]. (The main terminologies used in this study are summarized in Table 1).

**Table 1:** The terminologies used in this study.

Terminology	Explanation
Community or module	A set of nodes with denser links among them, but sparser with the rest of the network.
Overlapping community structure	
Cover of overlapping community structure	The overlapping community structure can be represented as a cover of network in which one node
	can belong to more than one community.
Overlapped node	A node can be shared by more than one community.
Non-overlapped node	A node only belongs to one community.
Non-overlapping community structure	
Partition of non-overlapping community structure	The non-overlapping community structure can be represented as a partition of network in which
	each node only belongs to one community.

### Regional nodal properties

We examined regional nodal properties of 90 brain regions in terms of the following metrics: node degree, nodal efficiency, and node betweenness. The node degree (*D*) of a node *i* is the number of connections that link it to the rest of the network. It is the most fundamental network measure, and most other measures are ultimately linked to it. The nodal efficiency (*E_nodal_*) for a given node *i* is defined as the inverse of the mean harmonic shortest path length between this node and all other nodes in the network [41], [43] and is defined by equation (4).
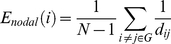
(4)The node betweenness centrality (*N_bc_*) of a node *i* is defined as the number of shortest paths between any two nodes that run through node *i* [44]. It is a widely used measure of a node's significance for the flow of information through the network. Finally, we defined the normalized metrics (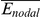,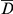,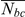) of a node as the ratio between the value of this node and the average value of all nodes. Moreover, we applied a simulated procedure to investigate how the robustness of the structural brain network is affected by the different types of lesions [40], [44], [45], [46]. We computed changes in both the global efficiency and the largest connected component size of the structural brain network in response to the continuous removal of the nodes (brain regions) in either random failures or targeted attacks by the decreasing node degree, nodal efficiency, node betweenness, and the participation coefficient in the non-overlapping partition, respectively (see Supplementary Text S2).

## Results

### Detecting overlapping community structure

**Figure 2:**
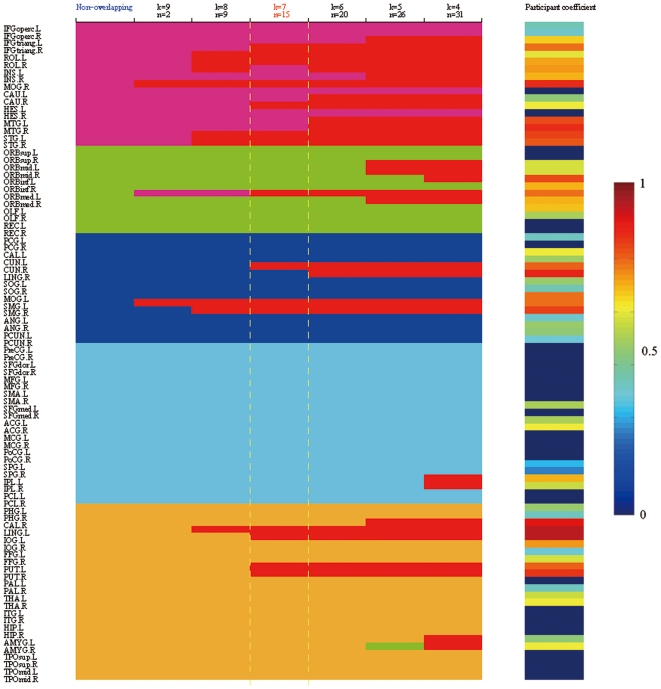
The dynamic processes of overlapping communities in the structural brain network. Using a fast unfolding algorithm on modularity optimization, both the overlapping community structure, setting parameter *k* to 4 through 9, and the non-overlapping community structure were obtained. Overlapping communities are painted with different colors (Community I: violet; Community II: green; Community III: blue; Community IV: cyan; Community V: orange). Overlapped nodes are painted red. The number of overlapped nodes is denoted by the parameter *n*. The participation coefficients of 90 regions in the non-overlapping community structure are plotted using the color bar (see supplementary Text S2). For a description of the abbreviations, see supplementary Table S1.

**Figure 3:**
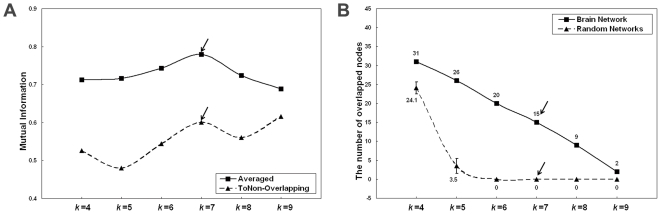
The selection for parameter *k*. (A) The mutual information of comparisons among the dynamic processes of the overlapping community structure of the structural brain network. Each point of the solid line indicates the mean value of mutual information by a specific value of *k*, which is averaged from the values of mutual information of comparisons between the cover represented by *k* and all other covers. Each point of the dashed line indicates the value of mutual information of the comparison between the cover of overlapping community structure by a specific value of *k* and the partition of non-overlapping community structure. (B) The number of overlapped nodes in the brain network (solid line) and random networks (dashed line). The number of overlapped nodes in random networks by each *k* value (mean ± sd) were obtained by 1000 matched random networks that preserve the same number of nodes, mean degree, and degree distribution as the brain network (Maslov and Sneppen 2002).

In this study, we thresholded the structural connection matrix into a binarized and undirected network *G* using a specific cost threshold (cost = 0.13), which resulted in the sparsest, fully-connected brain network. Unless stated otherwise, the results reported in this paper were mainly computed using this threshold. After we obtained the structural brain network, we applied the method proposed by Shen et al. (2009b) to detect overlapping community structures. In this analysis, the parameter *k* affects the constituent of overlapped nodes between communities. It is essential to select an appropriate value for the parameter *k*, although there is no criterion for the selection. The parameter *k* should not be too small because subordinate maximal cliques are not as highly connective. A larger value of *k* would result in a lower number of overlapped nodes (*n*). If *k* is large enough, the maximal clique network would be identical to the original network, and no overlap would be identified. In this study, we applied a range of *k* (*k* = 4–9) to calculate the covers of the overlapping community structure in the structural brain network. For the dynamic processes of overlapping communities in the structural brain network, only 2 overlapped nodes occurred when *k* was equal to 9, and 31 overlapped nodes were identified when the parameter *k* was decreased to 4 (Figure 2). We also computed a partition for the non-overlapping community structure in the structural brain network using the modularity optimization method [42], which was the same as the method of partitioning the maximal clique network. The structural brain network was separated into 5 non-overlapping communities (Figure 2). We then compared pairs among all covers with overlapping communities by mutual information, which is in the range [0,1] and equals 1 if and only if the two covers are equal [5]. Therefore, a larger value of mutual information indicates a higher similarity between two covers. We averaged the mutual information of pairs between overlapping covers, as shown in Figure 3A. Each point of the solid line indicates the mean value of mutual information by a specific value of *k*, which was averaged from the values of mutual information of comparisons between the cover represented by *k* and all other covers. The cover represented by *k* = 7 had the highest value, which revealed that this cover might be the most representative cover among all of the covers. We also compared the non-overlapping partition to all covers with overlapping communities (*k* = 4–9), as shown in Figure 3A. Each point of the dashed line indicates the value of mutual information of the comparison between the cover of the overlapping community structure represented by a specific value of *k* and the partition of the non-overlapping community structure. The covers created using the values *k* = 7 and *k* = 9 showed higher mutual information values, which implied that the partition with non-overlapping community structure was more similar to the cover with overlapping communities created using a value of *k* = 7 or *k* = 9. Using the same parameter *k*, we also calculated the number of overlapped nodes in 1000 matched random networks that preserve the same number of nodes, mean degree, and degree distribution as the brain network [47]. The overlapped nodes in the random networks disappeared when the parameter *k* was increased to 6 (Figure 3B). This result implied that overlapped nodes in the brain network show distinct topological properties compared to those in random networks. Therefore, we adopted *k* = 7 for the analysis of the overlapping community structure of the brain network.

### Overlapping communities

**Figure 4:**
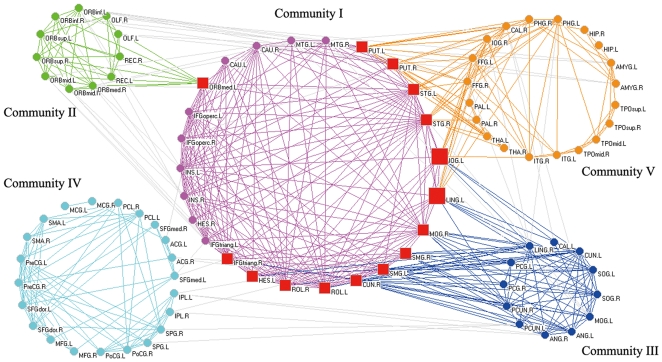
The topological representations of the overlapping community structure in the structural brain network. All 90 brain regions were organized into 5 overlapping communities painted with different colors (Community I: violet; Community II: green; Community III: blue; Community IV: cyan; Community V: orange). Overlapped nodes are indicated by square symbols (red colors), in which the large squares are shared by three communities, and the small squares are shared by two communities. Connections within the same community are painted with the color of the community. Connections between communities are painted with gray. For a description of the abbreviations, see supplementary Table S1.

**Figure 5:**
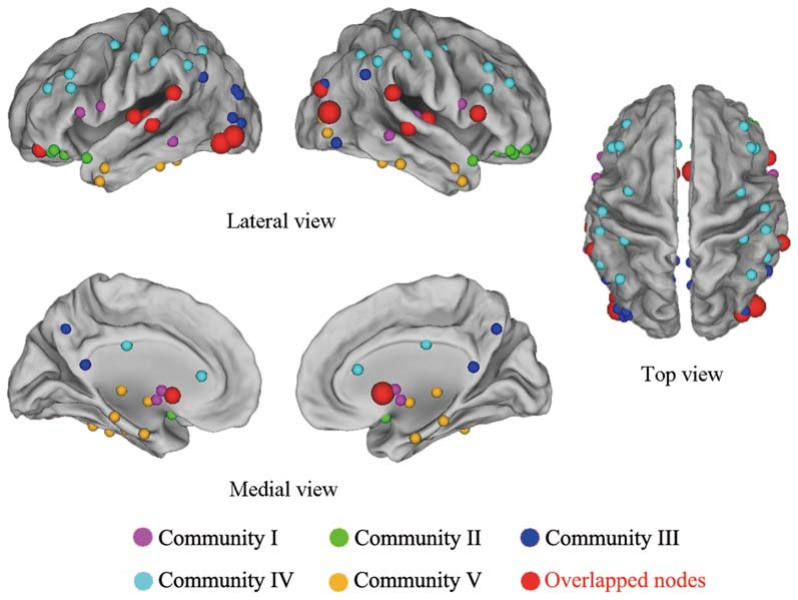
The surface representations of the overlapping community structure in the structural brain network. All 90 brain regions were organized into 5 overlapping communities painted with a different color (Community I: violet; Community II: green; Community III: blue; Community IV: cyan; Community V: orange). Overlapped nodes are indicated by red spheres, in which the large spheres are shared by three communities, and the small spheres are shared by two communities. Community I included all overlapped nodes, which were shared by at least one other community.

The structural brain network was separated into 5 overlapping communities (Table 2). The topological representation of the overlapping community structure in the structural brain network was drawn using the Pajek software package (http://vlado.fmf.uni-lj.si/pub/networks/pajek) (Figure 4). We also demonstrated the surface representation of the overlapping community structure in the structural brain network using the Caret software [48] (Figure 5). Community I included all of the 15 overlapped nodes and was designated as the “core” community in which the 25 brain regions identified were mostly found in the frontal lobe, the temporal lobe, the subcortex, and the occipital lobe. Community II included 1 overlapped node and was designated as the “prefrontal” community (preF community), in which all 12 regions were found in the prefrontal cortices. Community III included 10 overlapped nodes and was designated as the “occipital-parietal” community (O-P community), in which most of the 22 regions identified were found in the occipital lobe and the parietal lobe. Community IV was designated as the “frontal-parietal” community (F-P community) and had no overlapped nodes, with 14 regions located in the frontal lobe and 8 regions found in the parietal lobe. Community V included 6 overlapped nodes and was designated as the “temporal-occipital-subcortical” community (T-O-S community), in which 14, 6, and 6 regions were located in the temporal lobe, the occipital lobe, and the subcortical system, respectively. The separated communities of the structural brain network were illustrated in anatomical spaces in sagittal and top views (see supplementary Figure S1). For a detailed description of the constitution of overlapping communities, see supplementary Text S1.

**Table 2:** The overlapping community structure of the structural brain network.

Community	Name	Brain Function	Brain Regions	Overlapped Nodes	Lobe				
					Frontal	Temporal	Occipital	Parietal	Subcortical
I	Core	Auditory and language/visuospatial	25	15	7	6	4	2	6
II	Prefrontal	Emotion/decision-making	12	1	12				
III	O-P	Social/visual(DP)	22	10	3	1	10	8	
IV	F-P	Control of action	22	0	14			8	
V	T-O-S	Memory and learning/visual(VP)	26	6		14	6		6
Total			90	15	**32 (4)**	**18 (3)**	**14 (4)**	**16 (2)**	**10 (2)**

The cover of the structural brain network was obtained by *k* = 7 here. The number of brain regions (overlapped nodes) included in each lobe was indicated by the bold characters. O-P: Occipital-Parietal; DP: dorsal pathway; F-P: Frontal-Parietal; T-O-S: Temporal-Occipital-Subcortical; VP: ventral pathway.

### Overlapping nodes

**Figure 6:**
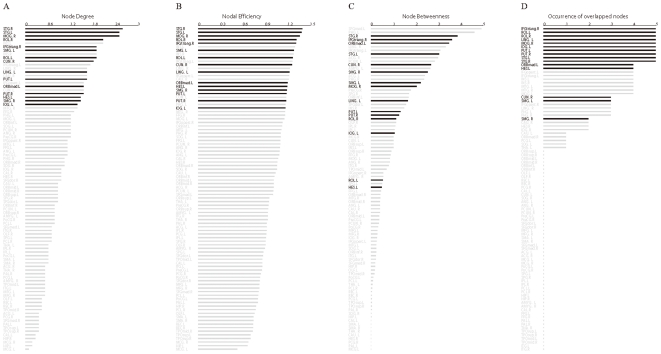
The regional nodal properties of overlapped nodes in the structural brain network. The bar plot of all 90 regions is listed in descending order of their (A) node degree, (B) nodal efficiency, and (C) node betweenness, respectively. (D) The occurrence of overlapped nodes in the structural brain networks constructed at all selected cost thresholds (cost = 0.13, 0.15, 0.18, 0.20, 0.22); the overlapping community structure of all brain networks was detected using the same parameter (*k* = 7). Regions with a high occurrence were determined to be insensitive to the selection of cost thresholds. The black and gray bars indicate overlapped nodes and non-overlapped nodes, respectively. For a description of the abbreviations, see supplementary Table S1.

**Figure 7:**
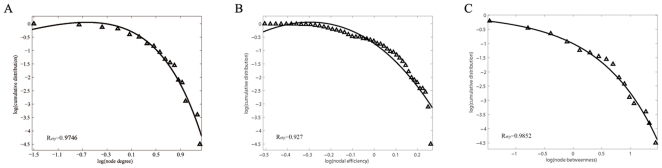
Topological distribution of the structural brain network. (A) Log-log plot of the cumulative probability of node degree distribution. (B) Log-log plot of the cumulative probability of nodal efficiency distribution. (C) Log-log plot of the cumulative probability of node betweenness distribution. The solid lines indicate the fits of the exponentially truncated power law [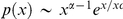]. R-squared values indicate the goodness of the fits.

**Figure 8:**
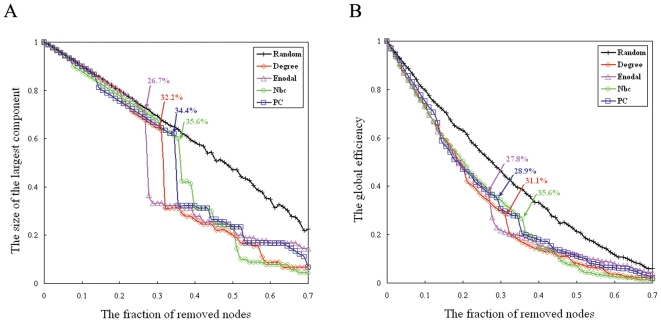
Network robustness in the structural brain network. (A) Changes in global efficiency as a function of the fraction of removed nodes in either random failures or targeted attacks caused by decreasing node degree, nodal efficiency, node betweenness, and the participation coefficient (in the non-overlapping partition). (B) Changes in the size of the largest component as a function of the fraction of removed nodes in either random failures or targeted attacks caused by decreasing node degree, nodal efficiency, node betweenness, and the participation coefficient (in the non-overlapping partition).

As indicated by the overlapping community structure, 15 regions were recognized as overlapped nodes; specifically, 11 association regions, 2 subcortical regions, 1 limbic/paralimbic region, and 1 primary region were recognized as overlapped nodes (Table 3). The overlapped nodes were mostly identified in an inferior-posterior pattern (Figure 5). We observed that the overlapped nodes might be primarily related to regions with higher values of node degree (top 25%) (Figure 6A) and nodal efficiency (top 26%) (Figure 6B). However, the node betweenness of the overlapped nodes was scattered (Figure 6C). We also found that most of the overlapped nodes were insensitive to the selection of cost thresholds; the results showed a higher occurrence of five cost thresholds (cost = 0.13, 0.15, 0.18, 0.20, 0.22) (Figure 6D). Moreover, we showed that the distribution of these nodal properties followed an exponentially truncated power law distribution model, implying a lack of nodes with extremely high values in these metrics (Figure 7). Furthermore, to assess the effects of nodal ‘lesions’ on the overall topology of the brain structural network, a simulation analysis was performed to examine the network robustness after individual nodes were continuously removed in a manner of random failure or targeted attacks. As expected, the continuous targeted attacks caused by decreased node degree, nodal efficiency, node betweenness, and the participation coefficient (in the non-overlapping partition) had a more dramatic effect on the brain structural network performance (the global efficiency and the size of the largest component) than the random failure of regions (Figure 8). For instance, when 26.7% of the regions with higher values of nodal efficiency (Figure 8A, violet arrow), 32.2% of the regions with higher values of node degree (Figure 8A, red arrow), or 34.4% of the regions with higher values of the participation coefficient (Figure 8A, blue arrow) were attacked in the brain network, the size of the largest component decreased sharply; in this case, all of the overlapped nodes were removed. This result demonstrated that overlapped nodes usually showed higher nodal efficiency and node degree and were highly related to those with high participation coefficients, which are usually defined as “connectors” in a non-overlapping community structure.

**Table 3:** The overlapped nodes in the structural brain network.

Region	Lobe	Class	Degree	Enodal	N_bc_	Community	Brodmann's Area			Reference
							U	M	T	
STG.R	Temporal	Association	2.59	1.39	3.86	I, V	41, 42	22		B, C, G
STG.L	Temporal	Association	2.50	1.36	2.04	I, V	41, 42	22		B, G
MOG.R	Occipital	Association	2.50	1.38	3.04	I, III	18	19		A, G
ROL.R	Frontal	Association	2.07	1.32	1.13	I, III			48	
IFGtriang.R	Frontal	Association	1.90	1.31	3.61	I, III		45		E, G
SMG.L	Parietal	Association	1.90	1.26	0.54	I, III		40		B
ROL.L	Frontal	Association	1.90	1.28	2.22	I, III			48	
CUN.R	Occipital	Association	1.81	1.25	2.68	I, III	17, 18	19	23	G
LING.L	Occipital	Association	1.64	1.22	1.65	I, III, V	17, 18	19		G
PUT.L	Subcortical	Subcortical	1.64	1.18	1.32	I, V			48	D
ORBmed.L	Frontal	Paralimbic	1.55	1.20	3.48	I, II		10	11	C
PUT.R	Subcortical	Subcortical	1.55	1.17	1.25	I, V			48	D, F
HES.L	Temporal	Primary	1.47	1.19	2.54	I, III	41, 42			
SMG.R	Parietal	Association	1.47	1.19	0.47	I, III		40		C
IOG.L	Occipital	Association	1.38	1.17	1.06	I, III, V	18	19, 37		

The brain regions were listed by a descending of their node degree. The regions are classified as association, primary, limbic/paralimbic or subcortical regions as described by [63]. R: right; L: left. For the description of the abbreviations, see Table S1. Brodmann's areas are categorized into unimodal (U), multimodal (M), or transmodal (T) divisions using the criteria described by [63]. The reference column indicates the hub regions previously identified in human brain structural (A, B, C, D, E) or functional (F, G) networks. A; Gong et al. (2009), B; He et al. (2008), C; Chen et al. (2008), D; Iturria-Medina (2008), E; He et al. (2007), F; He et al. (2009), G; Achard et al. (2006).

## Discussion

This is the first study to demonstrate an overlapping community structure in the structural brain network. The brain network was constructed by the measurement of RGMV in 462 young, healthy individuals. The division of 90 brain regions into 5 overlapping communities with functional significance suggested that the structural brain network reflects the functional organization of the human brain. The overlapped nodes shared by more than one community might be involved in different brain systems. We showed that the overlapped nodes revealed prominent regional nodal properties and played a pivotal role in the structural brain network. The overlapped nodes with a higher node degree and nodal efficiency mostly contributed to ventral frontal-temporal-occipital cortices, which are primarily related to auditory and visual perception and are likely to be early developed brain regions. Our results provide new insights into the understanding of the relationship between the structure and function of the human brain.

### Overlapping communities in the structural brain network

We proposed an overlapping community structure for the structural brain network in the human brain. The definition of overlapping community structure relies on the basic observation of a typical community consisting of several complete (fully connected) subgraphs that tend to share many of their nodes [6]. One node can participate in more than one community; therefore, overlapping communities naturally occur. Such an overlapping community structure can be represented by a cover of networks, and its identification in complex networks has been widely studied [5], [6], [8], [9], [10], [11], [49], [50]. In this study, the overlapping community structure was identified through partitioning the maximal clique network using the modularity optimization method [11].

In the cover with overlapping communities, our results demonstrated that the structural brain network was organized into 5 topological communities that corresponded to several well-known functional systems in the human brain. Previous studies on several real networks have shown that the detection of overlapped nodes as members of communities can be interpreted as a prediction of their functions [5], [6]. Thus, the analysis of brain functions within overlapping communities and related overlapped nodes is of great importance and significance (Table 2). Most of the regions in the “core” community (Community I) were associated with auditory and language/visuospatial functions. The characterization of this community was in agreement with many previous studies that identified the human language network and its structure-function relationship using fMRI and DTI [51], [52], [53]. This community included several regions in the occipital lobe (CUN.R, LING.L, MOG.R, and IOG.L) that are primarily associated with visuospatial processing and are found in verbal tasks involving visual/spatial relations [54], [55]. Moreover, the “core” community was also in accordance with a multimodal cortical network that is responsive to transitions in multiple sensory modalities (visual, auditory, and tactile stimuli) [23]. The regions included in Community II from the prefrontal cortex were mainly responsible for emotion and decision-making. This finding was in accordance with a previous study demonstrating that the orbitofrontal cortex represents a critical structure in a neural system that sub-serves decision-making [56]; it also supported the fact that many current theories on decision-making address emotion as a factor [57]. Regarding the two cortical pathways for visual perception, many regions in Community III from the occipital and parietal lobes (bilateral CUN, LING, SOG, MOG, ANG, PCUN, CAL.L, and IOG.L) were related to the dorsal pathway, which is specialized for determining “where an object is”; some regions in Community V (bilateral FFG, IOG, ITG, LING.L, and CAL.R) were associated with the ventral pathway, which is specialized for determining “what we're looking at” [58], [59]. Additionally, many regions in Community III, such as the 6 overlapped nodes shared by Community I (bilateral ROL, SMG, IFGtriang.R, and HES.L), were also found to be associated with the “C-system” of the social brain [60]. Many regions in Community V from the temporal cortex and subcortical areas, such as hippocampus, parahippocampal gyrus, and amygdala, were associated with the biological memory system and the learning of skills and habits [57], [61]. Most of the regions in Community IV participated in the control of actions involving motor planning, movement preparation, and movement execution [57], [62].

Our results revealed several higher-level circuits or systems that are involved in complex behaviors, such as auditory and visual perception and motor control, or cognitive processes, such as memory, language, and emotion. These findings are compatible with previous studies on the modular organization of structural and functional networks in the human brain. The modular organization of the structural brain network was first revealed by cortical thickness measurements from structural MRI analyses in which 45 cortical regions were organized into 6 topological modules (sensorimotor, auditory, visual, attention, and mnemonic processing) that closely overlap known functional domains [16]. He *et al.* reported that spontaneous brain function networks have an intrinsically cohesive modular organization in which the identified modules are found to be closely associated with several well-known, functionally interconnected subsystems, such as the somatosensory/motor, auditory, attention, visual, subcortical, and “default” systems [18]. Meunier *et al.* demonstrated that three major modules are recognized in human brain functional networks, including central (presumably motor and auditory/language), posterior (presumably visual), and dorsal fronto-cingulo-parietal modules (presumably attention and default-mode functions) [19].

### Overlapped nodes in the structural brain network

Our results revealed that overlapped nodes were shared by different communities (which represented brain systems). Most of the overlapped nodes were found to be involved in multimodal or transmodal cortices (Table 3), which provide anatomical and computational epicenters for large-scale neurocognitive networks [63]. These findings might provide evidence demonstrating that the human brain contains a system of multimodal areas. The cerebral cortex has been traditionally divided into separate territories for functions such as vision, touch, audition, and movement, which are known to overlap in many parts of the cortex. Bremmer et al. 2000 reported a major advance in understanding the regions of overlap in the human brain in which the senses are integrated [64]. There is recent electrophysiological and brain imaging evidence showing that visual, auditory, and somatosensory integration occurs in early stages of the visual cortical pathways; for example, this integration has been shown to occur around the lingual gyrus (an overlapped node shared by Community I, III, and V) where Brodmann's area 17 is located [65], [66]. The superior temporal gyrus (two overlapped nodes shared by Community I and V) and the supramarginal gyrus (two overlapped nodes shared by Community I and III) play an important role in the social brain [57], [60], although these regions are mainly responsible for auditory/language processing. Although the putamen (two overlapped nodes shared by Community I and V) has many functions because it is interconnected with many other structures, its main function is to regulate movement, influence various types of learning, and play a role in speech motor control [67], [68], [69].

We also noted that overlapped nodes were mostly attributed to ventral frontal-temporal-occipital cortices in an inferior-posterior pattern (Figure 5). These regions were primarily related to auditory and visual perception and are likely to develop early. The auditory and visual systems are two of the sensory modalities that have distinct cortical representations and provide information about the external environment for cognitive processing [70]. More interestingly, there was only one community (Community IV) without an overlapped node, which mainly participated in the control of action. These findings were in accordance with the results of a recent study showing that, in the infant brain, cortical hubs and their associated cortical networks are largely confined to primary sensory and motor brain regions and that the functional network architecture is linked to support tasks that are of a perception-action nature [71]. Our findings were also consistent with previous results showing that neurons in early sensory cortical areas are influenced by more than one modality and that multisensory processing begins in early cortical areas [72], [73], [74], [75], [76].

### Regional nodal properties

Our results demonstrated the topological importance of overlapped nodes, which revealed prominent regional nodal properties and played a pivotal role in the structural brain network. The overlapped nodes showed a higher node degree, nodal efficiency, and density connections. These brain regions were mostly in accordance with global hubs defined in previous studies on the structural and functional networks of human brains (Table 3) [16], [18], [28], [40], [46], [77], [78], [79]. Thus, overlapped nodes with a higher node degree and nodal efficiency should be of great importance for communication within the network and suggest a role for global hubs. However, distinct discrepancies between our results and previous studies were also observed due to differences in neuroimaging modalities, characteristics of subjects, and metrics for defining global hubs (such as the node degree, the nodal efficiency, the characteristic path length, and the node betweenness). To investigate the correlation between regional nodal properties, we computed the Pearson Coefficient of the comparisons among the three metrics adopted in this study. We demonstrated high accordance between node degree and nodal efficiency, whereas node betweenness had lower correlation to other metrics (see supplementary Figure S2). This result supported our finding that similar rankings of overlapped nodes were found between node degree and nodal efficiency. Moreover, we found that the overlapped nodes were also related to regions with a higher participation coefficient in the partition with non-overlapped nodes. Nodes with a higher value of the participation coefficient have much more inter-module (inter-community) connections in the non-overlapping community structure and are usually defined as “connectors” that play a critical role in the coordination of information flow over the whole network [18], [19]. Thus, the topological role for overlapped nodes was also similar to that of “connectors”, which are likely to be responsible for inter-module communication.

### Methodological limitations

**Figure 9:**
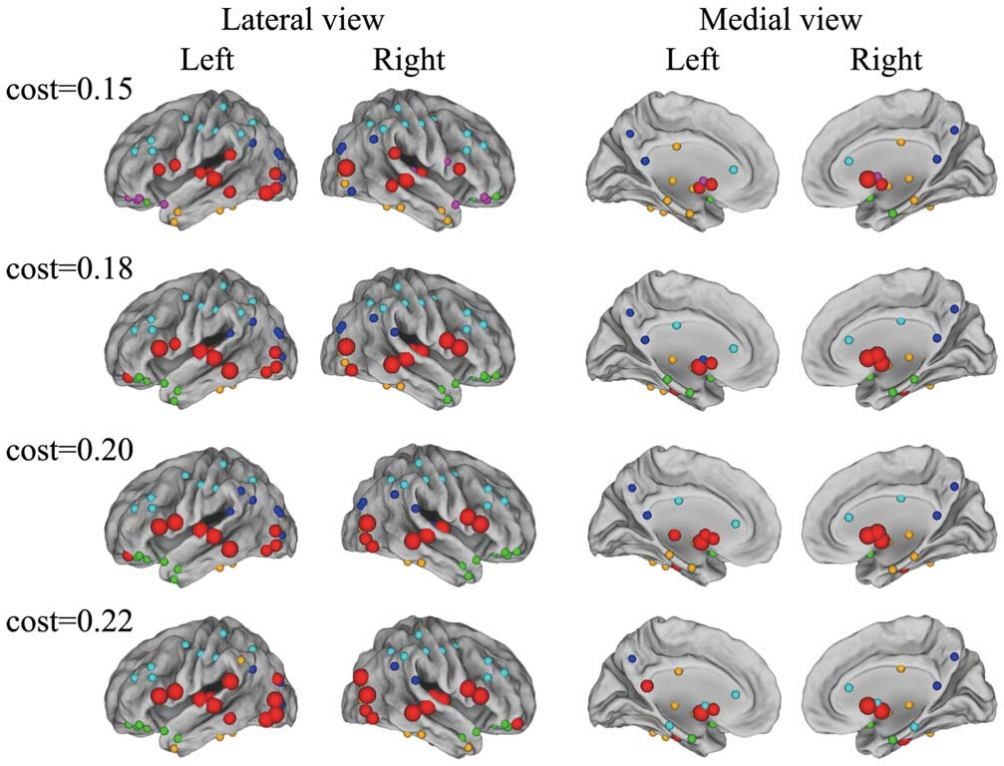
The overlapping community structure of structural brain networks constructed at multiple cost thresholds (cost = 0.15, 0.18, 0.20, 0.22). The analysis of the overlapping community structure was performed using the same parameter (*k* = 7). All 90 brain regions were organized into overlapping communities painted with different colors. Overlapped nodes are indicated by red spheres, in which the large spheres are shared by three communities, and the small spheres are shared by two communities.

Several methodological issues need to be addressed. First, in this study, we used the RGMV measurement to construct structural brain networks, as applied by a previous study on the hierarchical organization of human cortical networks [26]. Although there is no direct proof showing that correlations of gray matter volume across subjects are indicative of axonal connectivity via white matter tracts, strong correlations between brain regions that are known to be anatomically connected have been observed in previous optimized voxel-based morphometry studies [80], [81]. Moreover, the quantitative analyses of structural brain networks provide fresh insights into these questions [38]; for example, are there any other grey matter reductions accompanied by the atrophy of one brain region? Is age-related hippocampal degeneration related to degeneration elsewhere? What is the relationship between the atrophy of the prefrontal lobe with normal aging and atrophy of other cortical regions? Using RGMV as a measurement of structural connectivity is currently considered to be exploratory and should be investigated further in future studies. Second, different cost thresholds result in different numbers of edges in the brain network and may lead to different overlapping community structures. Thus, we applied multiple cost thresholds (cost = 0.15, 0.18, 0.20, or 0.22) to evaluate the stability of topological organization in the structural brain network. The cost thresholds were selected from the range (0.13≤cost≤0.25), which was adopted by the following complementary approaches: (1) all brain networks were fully connected, and (2) the resulting brain networks have sparse and distinguishable properties in comparison to degree-matched random networks [26], [82], [83]. We demonstrated the similar overlapping community structure in structural brain networks constructed at multiple cost thresholds (Figure 9). As the cost threshold increased, the number of edges in the brain network also increased, which resulted in an increase in overlapped nodes. Interestingly, the overlapped nodes were found to be mainly attributed to ventral frontal-temporal-occipital cortices and were involved in an inferior-poster pattern, suggesting a robust topological organization in the structural brain networks. Third, variations in parcellation templates (e.g., AAL used in this study) affect network structure in the human brain. A previous study indicated that regional volumes are positively correlated to their mutual information, which measures the functional connectivity between the region and the remaining brain regions [84]. Although gross inferences regarding network topology (e.g., small-world or scale-free) are robust to the template used, different parcellation strategies affect topological parameters (e.g., path length, clustering, small-worldness, and degree distribution) of structural or functional brain networks [27], [82], [85], [86]. Thus, the comparison of network parameters across studies must be made with reference to the spatial scale of the parcellation schemes. Moreover, because our results revealed the fuzzy boundaries between communities by identifying overlapped nodes, the overlapping community structure would be changed by parcellation templates due to different boundaries between brain regions. It would be worthwhile to identify the overlapping community structure with different parcellation templates in future studies. Fourth, while the majority of previously published works have adopted 1.5 T or 3 T MR scanners, the current findings were based on T1-weighted images using two 0.5 T MR scanners, which may lead to lower resolution of our results. Finally, while the binary brain network was analyzed in this study, it will be interesting to determine the overlapping community structure in weighted brain networks. Further investigations will also examine the overlapping community structure in the human brain network by different neuroimaging modalities, such as diffusion tensor imaging, functional MRI, and electroencephalography.

### Conclusions

In conclusion, the overlapping community structure was identified in the structural brain network derived from the measurement of RGMV in 462 young, healthy individuals. Overlapping communities were associated with known functional specializations of brain regions. Overlapped nodes were found in an inferior-posterior pattern and were mainly related to brain regions with a higher node degree and nodal efficiency, which played a pivotal role in the flow of informa- tion through the structural brain network. The identification of overlapping communities and overlapped nodes may provide valuable insights into the understanding of the structure and function of the human brain.

## Supporting Information

Figure S1 The anatomical representations in the sagittal (A) and top (B) view of each overlapping community in the structural brain network. The overlapping community structure was computed by parameter k = 7. The overlapped nodes are painted with more than one color (2 colors: shared by two communities; 3 colors: shared by three communities). Note that the figures in the top view were adjusted to the same size.
(DOC)

Figure S2 Comparisons of regional nodal properties in structural brain networks constructed by different cost thresholds. Each comparison between two nodal properties was evaluated by the Pearson Coefficient. (A) The results by cost thresholds equal to 0.13 and 0.18 are shown, respectively. The correlation between the nodal property and itself was set to zero. All Pearson Coefficients were significant at the 0.01 level (2-tailed). (B) Comparisons among three nodal properties were computed in each cost threshold under the range of 0.13∼0.30. The arrows indicate the results by cost thresholds of 0.13 and 0.18, respectively.
(DOC)

Table S1 Regions of interest included in AAL-atlas.
(DOC)

Text S1 The constitution of overlapping communities.
(DOC)

Text S2 The calculation of the participation coefficient in the non-overlapping partition.
(DOC)
