# Supplementary material for: The Overlapping Community Structure of Structural Brain Network in Young Healthy Individuals
Source: PLoS One. 2011 May 6;6(5):e19608. doi: 10.1371/journal.pone.0019608 (PMC3089616; doi:10.1371/journal.pone.0019608)
Supplement: Figure S1 — The anatomical representations in the sagittal (A) and top (B) view of each overlapping community in the structural brain network. The overlapping community structure was computed by parameter k = 7. The overlapped nodes are painted with more than one color (2 colors: shared by two communities; 3 colors: shared by three communities). Note that the figures in the top view were adjusted to the same size. (DOC) [file pone.0019608.s001.doc]

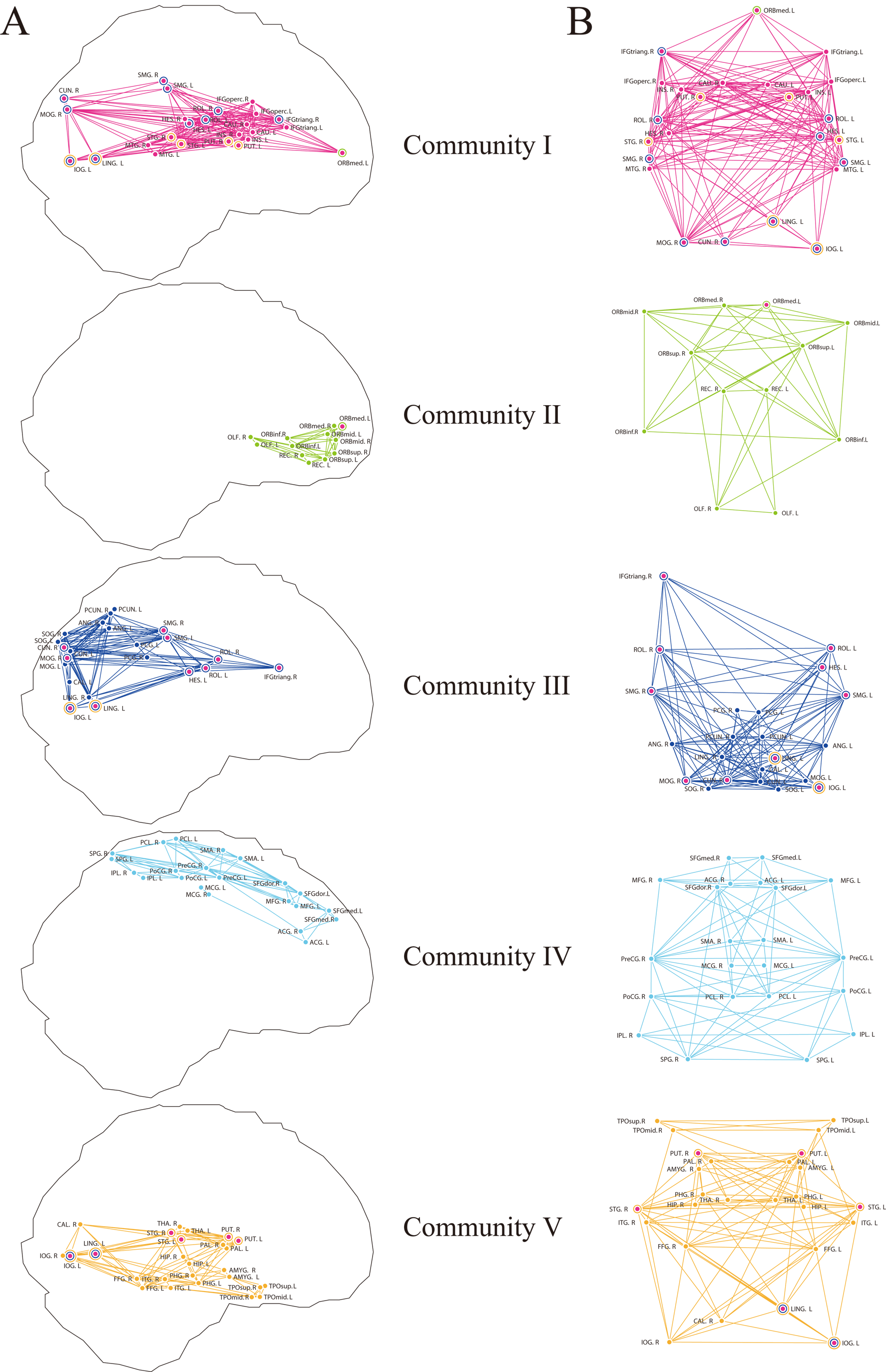


Figure S1 The anatomical representations in the sagittal (A) and top (B) view of each overlapping community in the structural brain network. The overlapping community structure was computed by parameter *k*=7. The overlapped nodes are painted with more than one color (2 colors: shared by two communities; 3 colors: shared by three communities). Note that the figures in the top view were adjusted to the same size.
