# Supplementary material for: The Overlapping Community Structure of Structural Brain Network in Young Healthy Individuals
Source: PLoS One. 2011 May 6;6(5):e19608. doi: 10.1371/journal.pone.0019608 (PMC3089616; doi:10.1371/journal.pone.0019608)
Supplement: Figure S2 — Comparisons of regional nodal properties in structural brain networks constructed by different cost thresholds. Each comparison between two nodal properties was evaluated by the Pearson Coefficient. (A) The results by cost thresholds equal to 0.13 and 0.18 are shown, respectively. The correlation between the nodal property and itself was set to zero. All Pearson Coefficients were significant at the 0.01 level (2-tailed). (B) Comparisons among three nodal properties were computed in each cost threshold under the range of 0.13∼0.30. The arrows indicate the results by cost thresholds of 0.13 and 0.18, respectively. (DOC) [file pone.0019608.s002.doc]

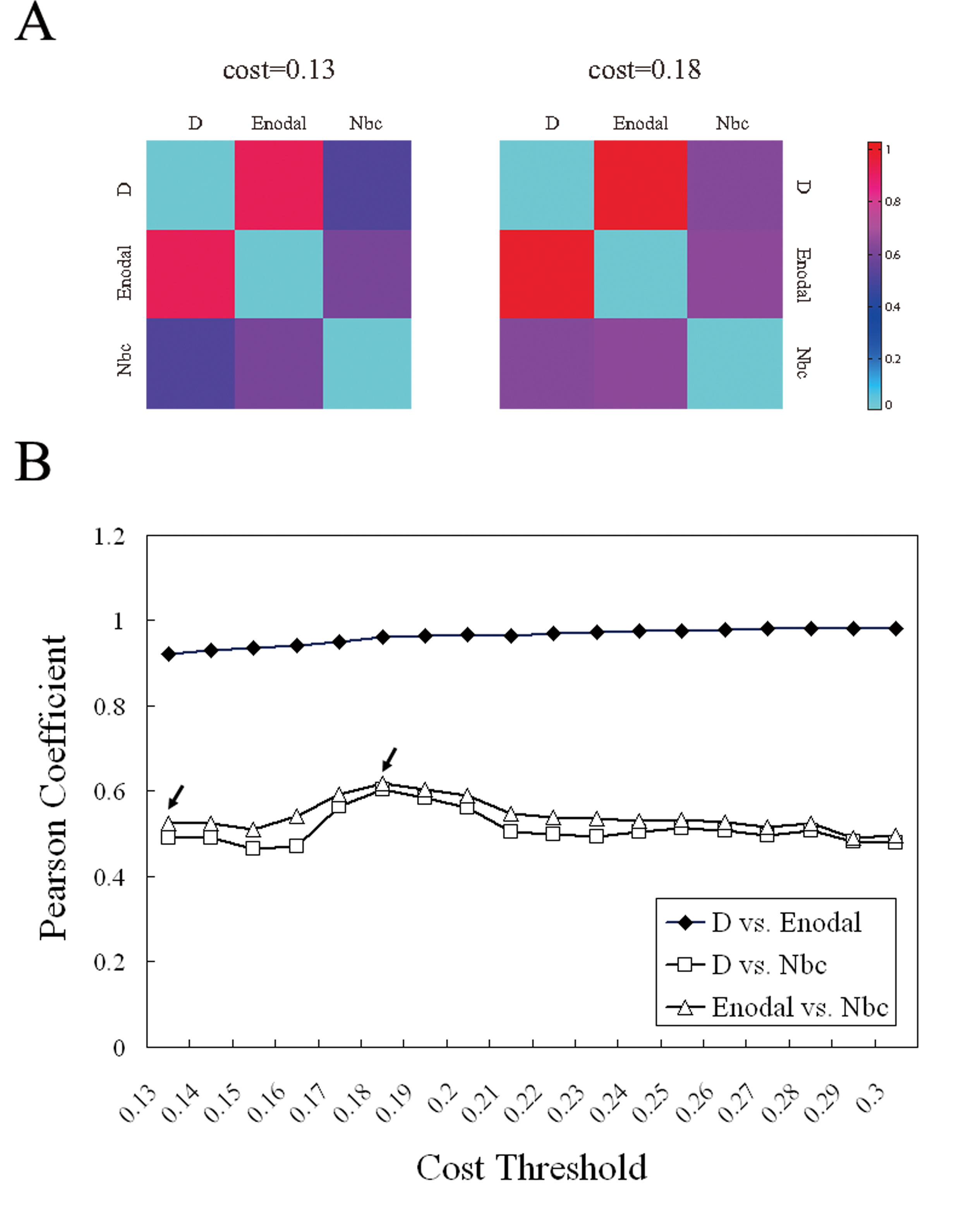


Figure S2 Comparisons of regional nodal properties in structural brain networks constructed by different cost thresholds. Each comparison between two nodal properties was evaluated by the Pearson Coefficient. (A) The results by cost thresholds equal to 0.13 and 0.18 are shown, respectively. The correlation between the nodal property and itself was set to zero. All Pearson Coefficients were significant at the 0.01 level (2-tailed). (B) Comparisons among three nodal properties were computed in each cost threshold under the range of 0.13~0.30. The arrows indicate the results by cost thresholds of 0.13 and 0.18, respectively.
