# Supplementary material for: The Overlapping Community Structure of Structural Brain Network in Young Healthy Individuals
Source: PLoS One. 2011 May 6;6(5):e19608. doi: 10.1371/journal.pone.0019608 (PMC3089616; doi:10.1371/journal.pone.0019608)
Supplement: Text S1 — The constitution of overlapping communities. (DOC) [file pone.0019608.s004.doc]

**Text S1 The constitution of overlapping communities**

Community I was designated as the “core” community and included 25 brain regions in the following locations: 7 regions of the frontal lobe, namely the left medial orbitofrontal cortex, bilateral inferior frontal gyrus (both opercular and triangular parts), and bilateral rolandic operculum; 6 regions of the temporal lobe, namely bilateral heschl, middle and superior temporal gyri; 6 regions of the subcortex (bilateral putamen, insula, and caudate); 4 regions of the occipital lobe (left inferior and right middle occipital gyrus, right cuneus, and left lingual gyrus); and 2 regions of the parietal lobe (bilateral supramarginal gyrus).

Community II was designated as the “prefrontal” community (preF community). All 12 regions were from the frontal lobe, namely bilateral orbitofrontal cortex (superior, middle, medial, and inferior), rectus gyrus, and olfactory cortex.

Community III was designated as the “occipital-parietal” community (O-P community). It included the following regions: 10 regions of the occipital lobe, namely occipital gyrus (bilateral superior and middle, left inferior), bilateral lingual gyrus, and bilateral cuneus; 8 regions of the parietal lobe (bilateral supramarginal gyrus, posterior cingulated gyrus, angular gyrus, and precuneus); 3 regions of the frontal lobe (bilateral rolandic operculum and right inferior frontal gyrus triangular part); and 1 region of the temporal lobe (left heschl gyrus).

Community IV was designated as the “frontal-parietal” community (F-P community). It consisted of 22 regions: 14 regions of the frontal lobe, namely bilateral precentral gyrus, superior frontal gyrus (dorsal and medial), middle frontal gyrus, supplementary motor area, and cingulate gyrus (middle and anterior); and 8 regions in the parietal lobe, namely bilateral postcentral gyrus, superior parietal gyrus, inferior parietal lobule, and paracentral lobule.

Community V was designated as the “temporal-occipital-subcortex” community (T-O-S community). It mainly included 26 regions: 14 regions of the temporal lobe, namely bilateral temporal gyrus (superior and inferior), hippocampus, parahippocampal gyrus, amygdala, and temporal pole (superior and middle); 6 regions of the occipital lobe (bilateral inferior occipital gyrus, right calcarine cortex, left fusiform gyrus, and left lingual gyrus); and 6 regions of the subcortical cortex (bilateral putamen, pallidum, and thalamus).
