# Supplementary material for: The Overlapping Community Structure of Structural Brain Network in Young Healthy Individuals
Source: PLoS One. 2011 May 6;6(5):e19608. doi: 10.1371/journal.pone.0019608 (PMC3089616; doi:10.1371/journal.pone.0019608)
Supplement: Text S2 — The calculation of the participation coefficient in the non-overlapping partition. (DOC) [file pone.0019608.s005.doc]

**Text S2 The calculation of the participation coefficient in the non-overlapping partition**

In the analysis of non-overlapping community structure, we also calculated the participation coefficient (*PC*) of nodes to distinguish the roles of nodes in terms of their intra-module (intra-community) and inter-module (inter-community) connectivity patterns [1,2]. The *PC(i)* of a node *i* is defined as

where *N_M_* is the number of modules, *k_is_* is the number of links of node *i* to nodes in module *s*, and *k_i_* is the total degree of node *i*. The *PC(i)* tends to 1 if node i has a homogeneous connection distribution with all the modules and to 0 if it does not have any inter-module connections. *PC* measures the ability of a node to maintain the communication between its own module and the other modules. A high *PC* value for a given node usually means the node has many inter-module connections. The participation coefficients of 90 brain regions are shown by the color bar (the right side, Figure 2). Results indicated that overlapped nodes were mainly related to regions with higher participation coefficient in the non-overlapping community structure. For instance, the overlapped nodes in the cover with overlapping communities by *k*=7 (the results described in main text) had higher participation coefficient (mostly in top 24%) in the non-overlapping community structure.

**Reference**

1. Guimerà R, Amaral LAN (2005) Functional cartography of complex metabolic networks. Nature 433: 895-900.

2. Guimerà R, Mossa S, Turtschi A, Amaral LAN (2005) The worldwide air transportation network: Anomalous centrality, community structure, and cities' global roles. Proc Natl Acad Sci USA 102: 7794-7799.
